# Effects of Isoflavone Intake on Energy Requirement, Satiety, and Body Composition of Neutered Adult Cats

**DOI:** 10.3390/ani14243574

**Published:** 2024-12-11

**Authors:** Ana Lúcia Yoshida da Silva Yamada, Mônica Estela Zambon Merenda, Layne Carolina Pereira, Nayara Maira Dalgallo Bonneti, Isabela de Oliveira Martins, Alina Stadnik Komarcheuski, Lucas Ben Fiuza Henríquez, Eduardo Kato Watanabe, Guilherme Bernardo Cornélio Coelho, Vanderly Janeiro, Nilva Maria Freres Mascarenhas, Ricardo Souza Vasconcellos

**Affiliations:** 1 Department of Veterinary Clinics, State University of Londrina (UEL), Londrina 86057-970, Brazil; 2 Department of Animal Science, State University of Maringá (UEM), Maringá 87020-900, Brazil; 3 Department of Agricultural Sciences, Ingá University Center (UNINGÁ), 6114 Highway PR317, Maringá 87035-510, Brazil; 4 Anestall, Maringá 87014-200, Brazil; 5 Sinus Vet, Maringá 87015-180, Brazil; 6 Department of Statistics, State University of Maringá (UEM), Maringá 87020-900, Brazil

**Keywords:** aglycones, daidzein, felines, genistein, nutrition

## Abstract

Isoflavones are composed of phytoestrogens (genistein and daidzein), which can be metabolized by cats. These compounds can promote the maintenance of lean body mass and control food intake. These effects are desirable in neutered animals, as they are predisposed to obesity. The objective of this study was to evaluate the effects of dietary supplementation of 1.0% isoflavone on the metabolizable energy intake, serum concentrations of satiety-related hormones and peptides, and body composition of neutered cats. Sixteen neutered adult cats were blocked by gender and divided into two groups (n = 8): the control group (CG) received a commercial diet, while the isoflavone group (IG) received the same diet supplemented by 1% of isoflavone for 99 days. Computed tomography was performed on the first and last experimental days to assess the animals’ body composition. Satiety challenges were conducted on days 19 and 44. In the last day of the study, blood samples were collected to determine the concentration of insulin, ghrelin, leptin, peptide YY, and GLP-1. A statistical analysis was conducted using R software 3.5.2, considering both the interaction and individual effects of group and time (*p* < 0.05). The average intake of genistein in the IG was 0.75 ± 0.10 mg/kg body weight, and daidzein intake was 51.73 ± 7.05 mg/kg. No significant individual or interaction effects were observed for any of the analyzed variables. Therefore, the inclusion of 1.0% isoflavone in the diet did not affect the energy requirements, satiety responses, or body composition of neutered adult cats.

## Simple Summary

The objective of this study was to assess the effects of supplementing 1.0% of isoflavone in a dry extruded diet for neutered adult cats on energy requirements, body composition, and satiety. Isoflavones are natural compounds found in certain plant products, such as soybeans. Previous research has indicated that these compounds may reduce body fat, decrease caloric intake, and enhance satiety. However, in this study, the administered dose did not lead to significant changes in the evaluated variables in domestic felines.

## 1. Introduction

Isoflavones are naturally present in different plants, primarily in legumes such as soybeans and their derivatives [1]. These phenolic compounds exist in two main forms: aglycones and glucosides. Aglycones are more lipid soluble compared to glucosides, facilitating their intestinal absorption and resulting in greater bioavailability and bioactivity in the gastrointestinal tract [2].

Within the group of aglycones, the two main molecules are genistein and daidzein, which share structural similarities with estradiol (17-β-estradiol). Although these compounds have low affinity for estrogen receptors compared to estradiol (α-ER and β-ER), they can bind to these receptors and induce estrogenic and antiestrogenic effects in mammals [3,4,5]. Additionally, some of their metabolites, such as equol, exhibit a high affinity for estrogen receptors [6,7]. These compounds are also associated with increased energy expenditure due to their ability to stimulate the coactivator PGC-1β, which enhances the expression of genes involved in fatty acid oxidation and mitochondrial activity in muscle cells [8].

Commercial diets for cats naturally contain these molecules (up to 154 mg genistein and 147 mg daidzein per kilogram of dry matter in dry food) due to the inclusion of soybean-derived ingredients such as isolated protein, concentrated protein, and soybean oil in diet formulations [6,9,10]. Furthermore, these compounds and their metabolites have been detected in the bloodstream of felines, confirming their absorption [6,11,12].

The effects of isoflavone consumption in mammalian species are diverse, including alterations in lipid and carbohydrate metabolism [13,14]. In humans, isoflavones have shown anti-inflammatory effects [15] and potential benefits in preventing cardiovascular diseases [16]. Moreover, in some species, genistein and daidzein exhibit anti-obesity effects [17,18]. Naaz et al. [19] investigated the effects of isoflavone supplementation in rodents, observing a decrease in adipose tissue fat deposition and a reduction in the expression of lipoprotein lipase, an enzyme involved in lipid uptake by adipocytes.

Neutering of companion animals induces metabolic and hormonal changes that predispose them to obesity. Neutered cats are more likely to become obese compared to intact ones [20,21]. Obesity is a multifactorial condition that compromises longevity and quality of life, and it is often associated with comorbidities such as dermatitis, diabetes mellitus, neoplasia, and urolithiasis in domestic felines [22].

Considering that domestic cats can absorb and metabolize phytoestrogens such as genistein and daidzein, the hypothesis of this study was that the inclusion of isoflavones from soybean, as a source of phytoestrogens, in the diet for neutered cats can improve body composition and promote satiety, potentially minimizing the negative effects associated with neutering. Given this, this study aimed to evaluate the effects of 1% isoflavone supplementation in neutered adult cats on caloric intake for weight control, satiety, body composition, and postprandial hormonal responses.

## 2. Materials and Methods

The experimental procedures were approved by the Ethics Committee on the Use of Animals (CEUA) from the State University of Maringá, Brazil (protocol number 1757010419).

### 2.1. Animals and Diets

Sixteen adult cats (4 years old), mixed breed, neutered, and healthy were used. Prior to the beginning of the study, all animals underwent a clinical evaluation, including physical examination, complete blood count, and biochemical tests. All animals had an ideal body condition score (5/9) [23].

The animals (6 males and 10 females) were blocked by gender and divided into two experimental groups, each comprising 3 males and 5 females. The control group (CG) received a standard commercial diet (Matisse Neutered Cats—Chicken, Farmina Pet Foods, Bragança Paulista, SP, Brazil) (Table 1). The isoflavone group (IG) was fed the same basal diet supplemented with 1.0% isoflavone. The isoflavone, along with 3% poultry fat and 1% liquid palatant for cats, was incorporated into the diet by coating. To ensure consistency in dietary composition, these ingredients were also added to the CG diet, replacing isoflavone with starch.

The isoflavone was sourced from Galena Química e Farmacêutica Ltda., Campinas, SP, Brazil, with analyzed levels of bioactive compounds 0.55% genistein and 37.62% daidzein.

The daily amount of food provided to each animal was calculated using the following equation: 75 × body weight^0.67^ [24]. Throughout the adaptation and experimental period, the quantity remained the same and was not adjusted. Food intake was monitored daily, and animals were weighed weekly. Food was provided twice daily (8 a.m. and 3 p.m.), and water was available ad libitum. In order to assess changes in body weight and caloric intake of the animals, the data were grouped into four periods: days 1 to 24, 25 to 48, 49 to 72, and 73 to 97.

### 2.2. Experimental Design

The experimental design adopted was a randomized block (gender) design with repeated measures over time. Prior to the commencement of the study, all animals underwent a 14-day period consuming the control diet for standardization of feeding conditions. Following this period, baseline blood samples (day 0) were collected to determine concentrations of insulin, ghrelin, leptin, peptide YY, and GLP-1. Additionally, computed tomography (CT) was conducted to evaluate body composition.

**Figure 1:**
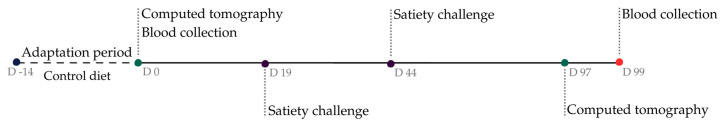
Experimental design.

Subsequently, the IG animals began consuming the isoflavone-supplemented diet, while the CG animals continued consuming the control diet for 99 days. Satiety challenges were conducted on days 19 and 44. On day 97, CT was performed again, and on day 99, blood samples were collected to determine hormone concentrations (Figure 1).

### 2.3. Computed Tomography

CT scans were performed at the beginning and end of the experimental period (days 0 and 97) using a helical tomograph (GE Hi Speed, Chicago, IL, USA). The animals were anesthetized to prevent movement during the CT scan. Pre-anesthetic medications and induction were administered intravenously: Fentanyl (3 µg/kg), Midazolam (0.2 mg/kg), Ketamine (2 mg/kg), and Propofol (4 mg/kg). Anesthesia maintenance was conducted using Isoflurane with a universal vaporizer at an oxygen flow rate of 2 L/kg/h, and parameters were monitored with a multiparameter monitor (MEC-1200 Mindray, Shenzhen, China).

**Figure 2:**
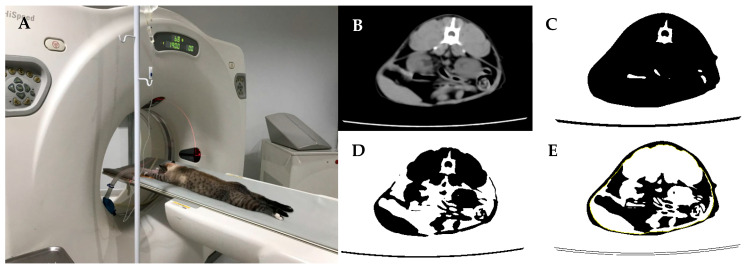
(**A**) The animal positioned in sternal recumbency for computed tomography imaging; (**B**) cross-section at lumbar vertebra; (**C**) Hounsfield Units (HU) histogram between −250 and +250, where black represents adipose and non-adipose tissue; (**D**) HU histogram between 0 and +250, where black represents lean mass; (**E**) HU histogram between −250 and 0, where black represents total fat and yellow represents subcutaneous fat.

The animals were positioned in sternal recumbency, and the scans were conducted in a cranial–caudal direction, encompassing the entire body, with a slice thickness of 7 mm (120 kV, 140 mA). The abdominal scans (between lumbar vertebrae 1 and 7) were performed with a slice thickness of 2 mm (120 kV, 115 mA) (Figure 2). The CT images obtained were analyzed using multimodality processing software (AW Volume Share 5—GE Healthcare, Chicago, IL, USA).

To assess body composition, scans at lumbar vertebra 2 (L2) were evaluated. The total percentage of adipose tissue was determined by excluding surrounding air and defining a histogram using Hounsfield Units (HU) limits between −250 and +250. Subsequently, the area represented by subcutaneous fat was excluded, and a new histogram was created with HU limits between −250 and +250 to identify intra-abdominal fat. This approach enabled the determination of percentages of total body, visceral, and subcutaneous adipose tissue at the L2 region (Image 3) [25,26]. The images were processed using ImageJ software (version 1.52r, Java image, Oracle Corporation, Austin, TX, USA).

### 2.4. Satiety Challenges

On days 19 and 44, at the time of the first meal (8 a.m.), the full daily amount of food was provided to the animals. Four hours later, the satiety challenge began, where a highly palatable food was offered to the cats (N&D grain free, Farmina Pet Foods, Bragança Paulista, São Paulo, Brazil). The label composition of the food was crude protein (minimum) 400 g/kg; ether extract (minimum) 200 g/kg; crude fiber (maximum) 22 g/kg; ash (maximum) 85 g/kg; moisture (maximum) 90 g/kg; and metabolizable energy 4200 kcal/kg. The amount of food provided in each satiety challenge was calculated by adding 25% to the average daily food intake per animal, resulting in a total of 70 g per cat. The food was available ad libitum for 5 h. Leftovers were weighed every hour, and consumption data were considered (adapted from [27]).

### 2.5. Hormone Concentrations

To determine the plasma concentration of insulin, ghrelin, leptin, peptide YY, and GLP-1, Milliplex/Millipore kits were used, employing xMAP^®^ technology (Luminex Corporation, Austin, TX, USA) standardized for the feline species.

To reduce the number of venipunctures and stress from serial blood collections, on day 97, double-lumen central catheters (4Fr × 5 cm—KFF S.A) were inserted into the jugular vein and secured with two simple interrupted sutures. To maintain the catheters and prevent clot formation, a heparin solution (0.5%) was used for flushing. The catheters were inserted into the animals after the CT scans while they were still anesthetized.

On day 99, blood samples were collected via the catheter at fasting (0 h) and at 1, 2, 4, and 6 h post-feeding. At each time point, 2 mL of blood was drawn into EDTA-coated tubes. Subsequently, 20 µL of Pefabloc^®^ SC PLUS (Roche, Basel, Switzerland) and 20 µL of DPP-IV Inhibitor (Millipore, Lenexa, KS, USA) were added to each sample to enable the measurement of ghrelin and GLP-1, respectively. After gently mixing by inverting ten times, the samples were centrifuged at 3000 rpm for 10 min. Plasma samples were subsequently transferred to Eppendorf tubes and stored at −20 °C until the day of analysis.

### 2.6. Statistical Analysis

For the statistical analysis, R software version 3.4.3 (R Core Team, 2018) was used along with the packages ExpDes [28] and Car [29]. Initially, Mauchly’s test was utilized to assess the sphericity of the data. If the result was positive (*p* > 0.05), the interactions and individual effects of group and time variables were analyzed. Mean differences were evaluated using Tukey’s test. Specifically for caloric intake data, a mixed-effects model analysis was conducted using the nlme package [30]. A significance level of *p* < 0.05 was adopted.

## 3. Results

All animals remained healthy throughout the study and exhibited sufficient food intake for maintenance, with no cases of vomiting or diarrhea. There was no difference between treatments in the animals’ body weight and caloric intake over time (Table 2 and Table 3).

No differences were observed in body composition between treatments throughout the study (Table 4).

In Table 5, the data obtained during the satiety challenges are presented. These data represent the amount of food (in grams) consumed during the challenges for each experimental group over the 5 h period.

Four animals from each group did not adapt to the catheters and had to be removed from the study on day 98, leaving only four animals per group for blood collection. Plasma concentrations of ghrelin, GLP-1, insulin, leptin, and peptide YY showed no difference between treatments (Table 6).

## 4. Discussion

Genistein and daidzein are natural compounds belonging to the group of isoflavones. These compounds can assist in lean mass gain, weight loss, and satiety in different species [31,32]. However, in this study, no difference in body weight was observed between the groups. On the other hand, an increase in caloric intake over time was noted regardless of group. A possible explanation for this result could be the temperature variation that occurred during the experimental period. Although the animals were housed in an indoor environment, the average temperature in the region where the facilities were located was 21.3 ± 4.4 °C. During periods of lower temperatures, felines tend to increase food intake due to the higher energy expenditure required to maintain body temperature (adaptive thermogenesis) [33].

In this study, the average consumption of genistein by the IG was 0.75 ± 0.10 mg/kg of body weight, and daidzein was 51.73 ± 7.05 mg/kg of body weight. Cave et al. [34] observed that administering 100 mg/kg of body weight of genistein to neutered adult cats (2 to 5 years old) reduced food intake during the experimental period. However, when the same dose was given to growing cats (12 to 16 months old), the authors did not observe an effect of genistein on satiety, suggesting potential age-related differences in the intestinal absorption or hepatic metabolism of genistein [31,34].

In cats, it has been observed that the bioavailability of genistein is lower when administered orally in powder form (100 mg/kg) compared to intravenous application (20 mg/kg). Nevertheless, the half-life of genistein in cats appears to be longer (17 h) than in other mammals, such as rats (2.71 h) and human beings (8 h) [11,35].

Although most studies on cats and other species focus on the effects of genistein in controlling weight and food intake, daidzein may also influence these parameters. In a study with obese rats, administering 50 mg/kg of body weight of daidzein for 13 days resulted in a reduction in both weight gain and caloric intake [17].

Fernandez-Garcia et al. [32] evaluated the daily administration of genistein (10 and 50 µg/g or 10 and 50 mg/kg body weight) in growing rats (2 weeks old) and found no isoflavone effect on caloric intake. The authors observed a gender effect, with males showing higher food intake and greater weight gain. Nevertheless, this effect was not observed in this study, nor has it been demonstrated in previous research on cats [34].

Due to the ability of isoflavones to interact with estrogen receptors and the coactivator PGC-1β, these compounds can modify body composition by altering adipocyte metabolic activity or modulating the action of hormones (ghrelin, leptin, and insulin) related to satiety [3,8,36,37]. In young female rats, dietary supplementation with genistein at increasing doses (0, 300, 500, 1000, and 1500 mg/kg) led to dose-dependent reductions in adipose tissue weight compared to the control group, with effects becoming more pronounced starting at 500 mg/kg [19]. In in vitro studies using isolated rat adipocytes, genistein and daidzein were found to operate through the AMPK/SREBP-1 pathway, inhibiting acetate conversion into lipids, reducing lipogenesis, and increasing lipolysis, thus decreasing lipid accumulation in these cells [38,39]. Despite this, in this study, the supplementation of isoflavones did not alter the body composition of neutered cats, and no differences in fat concentration and lean mass were observed over time. On the other hand, Cave et al. [31] observed an increase in lean mass over time in neutered cats given 100 mg/kg of genistein, indicating a physiological alteration in adipogenesis.

The regulation of food intake and satiety in companion animals is complex and involves various physiological processes. It can be influenced by factors beyond homeostasis and hormonal control, such as the addition of additives and fibers to the diet [34,40]. In this study, an adapted protocol from Bosch et al. [27] was used to evaluate the effect of isoflavone on the satiety of cats. The protocol involves providing the animals with highly palatable food a few hours after being fed the test diets.

On the days of the palatability challenges, caloric intake increased in both groups, with a 28.9% increase in the CG and 31.2% in the IG. Despite this, no difference was observed between the groups. However, a time effect was noted, with consumption progressively increasing during the 5 h the food was available.

The changes in satiety induced by neutering in cats have not yet been well elucidated. It has been observed that in males, food intake increases within a few days after neutering when food is provided ad libitum [41]. In female and male cats, neutering has been shown to reduce the maintenance energy requirements [42]. Therefore, the most effective approach to preventing excessive weight gain in neutered animals is through controlling the amount of food provided and monitoring the body condition score of the animals [41,43,44].

The release of hormones and peptides from the intestine and pancreas, which regulate satiety and energy expenditure, can be directly influenced by nutrients present in the intestinal lumen [45]. In this study, hormonal assays (ghrelin, GLP-1, insulin, leptin, and peptide YY) revealed no differences between groups. However, insulin and leptin concentrations increased over time.

Leptin, primarily synthesized by adipose tissue, plays a crucial role in regulating the organism’s energy status and modulating both short-term and long-term food intake by acting on the hypothalamus to increase satiety [46]. The glucose metabolism mediated by insulin in adipose tissue may explain the relationship between circulating leptin and insulin concentrations [47,48]. Insulin has been reported to stimulate leptin secretion in rat adipocytes by promoting glucose entry into the glucosamine pathway via L-glutamine: D-fructose-6-phosphate aminotransferase [49,50]. In rats, the administration of 1 g/kg of isoflavone in the diet can influence serum concentrations of insulin and leptin, leading to their reduction [36].

## 5. Conclusions

The inclusion of 1% isoflavone in the diet did not alter food intake, satiety, or body composition in neutered adult cats. Additionally, no differences were observed in the concentrations of hormones related to satiety. In future studies, it would be beneficial to explore the use of additional doses of isoflavones or their compounds (genistein and daidzein) to evaluate their effects in felines. Moreover, assessing their impact on obese animals or animals with unrestricted feeding could provide further insights into the action of these compounds in feline physiology.

## Figures and Tables

**Table 1:** Chemical composition of the control diet.

	Control Diet
Dry matter (%)	90.94
	**Chemical composition on DM basis (%)**
Organic matter	93.41
Crude protein	46.27
Fat	12.64
Ash	6.59
Crude fiber	1.73
Nitrogen-free extract	17.13
Gross energy (kcal/kg)	5265.57
Metabolizable energy (kcal/kg)	4098.05

Ingredient composition declared on the label: poultry by-product meal, broken rice, maize, sorghum, maize gluten, pea hulls, cellulose, fish meal, chicken fat, beet pulp, fish oil, dried egg, wheat bran, brewer’s dried yeast, DL-methionine, taurine, L-carnitine, chicken and pork liver hydrolysate, vitamins (A, D3, E, B1, B2, B6, B12, C, biotin, niacin, pantothenic acid, folic acid, and choline chloride), sodium chloride, potassium chloride, acidifying additive, ferrous sulfate, copper sulfate, zinc sulfate, manganese sulfate, sodium selenite, calcium iodate, cobalt sulfate, calcium propionate, BHA, and BHT. Legend: DM = dry matter

**Table 2:** Body weight (kg) of neutered adult cats receiving or not a basal diet supplemented with 1.0% isoflavone.

Group	Time (Days)				Mean	SEM	*p*-Value		
	1–24	25–48	49–72	73–97			Group	Time	GxT
**Control**	3.73	3.77	3.81	3.83	3.790	0.027	0.477	0.967	0.781
**Isoflavone**	3.61	3.68	3.72	3.71	3.680				
**Mean**	3.67	3.75	3.76	3.76					

Legend: SEM = standard error of the mean; GxT = interaction between Group and Time.

**Table 3:** Metabolizable energy intake (kcal/body weight^0.67^) of neutered adult cats receiving or not a basal diet supplemented with 1.0% isoflavone.

Group	Time (Days)				Mean	SEM	*p*-Value		
	1–24	25–48	49–72	73–97			Group	Time	GxT
**Control**	71.36	79.46	84.36	79.02	78.50	1.660	0.073	<0.0001	0.085
**Isoflavone**	67.76	72.69	79.10	76.67	74.01				
**Mean**	69.41 ^c^	75.93 ^b^	81.35 ^a^	78.64 ^ab^					

Legend: SEM = standard error of the mean; GxT = interaction between Group and Time; ^a, b, c^ means in the same line with common letters did not differ according to the Tukey test.

**Table 4:** Body composition of neutered adult cats receiving or not a basal diet supplemented with 1.0% isoflavone.

Group	Time (Days)		Mean	SEM	*p*-Value		
	0	97			Group	Time	GxT
**Total body adipose tissue (%)**							
**Control**	24.81	22.88	23.85	2.732	0.105	0.461	0.574
**Isoflavone**	20.16	22.38	21.27				
**Mean**	22.49	22.63					
**Intra-abdominal adipose tissue (%)**							
**Control**	22.51	18.84	20.68	2.495	0.091	0.176	0.829
**Isoflavone**	17.64	18.34	17.99				
**Mean**	20.08	18.59					
**Subcutaneous adipose tissue (%)**							
**Control**	2.30	4.04	3.17	0.551	0.441	0.0001	0.432
**Isoflavone**	2.52	4.04	3.28				
**Mean**	2.41	4.04					

Legend: SEM = standard error of the mean; GxT = interaction between Group and Time.

**Table 5:** Food consumption (grams) during satiety challenges of neutered adult cats receiving or not a basal diet supplemented with 1.0% isoflavone.

Group	Time (h)					Mean	SEM	*p*-Value		
Day 19	1	2	3	4	5			Group	Time	GxT
**Control**	22.50	28.88	32.63	37.13	41.00	32.42	1.187	0.534	<0.0001	0.116
**Isoflavone**	26.50	33.25	36.38	38.75	40.00	34.97				
**Mean**	24.50 ^d^	31.06 ^c^	34.50 ^b^	37.94 ^a^	40.50 ^a^					
**Day 44**	**1**	**2**	**3**	**4**	**5**	**Mean**	**SEM**	**Group**	**Time**	**GxT**
**Control**	18.13	23.50	28.75	29.50	35.00	26.97	1.540	0.215	<0.0001	0.591
**Isoflavone**	25.88	29.00	34.88	37.50	42.50	33.95				
**Mean**	22.00 ^d^	26.25 ^c^	31.81 ^b^	33.50 ^b^	38.75 ^a^					

Legend: SEM = standard error of the mean; GxT = interaction between Group and Time; ^a, b, c, d^ means in the same line with common letters did not differ according to the Tukey test.

**Table 6:** Fasting and postprandial hormone concentration (pg/mL) of neutered adult cats receiving or not a basal diet supplemented with 1.0% isoflavone.

Group	Time (h)					Mean	SEM	*p*-Value		
	0 (Fast)	1	2	4	6			Group	Time	GxT
**Control**	301.30	360.00	279.20	335.50	413.30	337.84	27.399	0.555	0.028	0.275
**Isoflavone**	231.80	228.80	282.30	379.30	361.50	296.70				
**Mean**	266.55 ^b^	294.37 ^ab^	280.72 ^b^	357.37 ^ab^	387.37 ^a^					
**Control**	60.18	82.26	75.23	79.37	108.82	81.17	12.937	0.719	0.094	0.072
**Isoflavone**	53.24	62.93	100.51	76.20	62.29	71.03				
**Mean**	56.71	72.59	87.87	77.78	85.55					
**Control**	5164.00	5257.00	4751.75	5866.25	7000.50	5607.90	511.615	0.207	0.012	0.677
**Isoflavone**	3738.75	3492.25	3627.75	4782.00	4628.00	4053.75				
**Mean**	4451.37 ^b^	4374.62 ^b^	4189.75 ^b^	5324.12 ^ab^	5814.25 ^a^					
**Control**	256.75	276.00	255.50	249.25	266.25	260.75	22.853	0.888	0.651	0.365
**Isoflavone**	234.00	248.00	258.25	267.50	259.25	253.40				
**Mean**	245.37	262.00	256.87	258.37	262.75					
**Control**	8.66	29.93	20.59	17.19	17.40	18.75	4.390	0.994	0.163	0.253
**Isoflavone**	9.82	13.82	21.69	27.00	21.77	18.82				
**Mean**	9.24	21.87	21.14	22.09	19.58					

Legend: SEM = standard error of the mean; GxT = interaction between Group and Time.; ^a, b^ means in the same line with common letters did not differ according to the Tukey test. For these variables, only 4 animals per treatment were used.

## Data Availability

The original contributions presented in the study are included in the article; further inquiries can be directed to the corresponding author.
